# Cultural vs. State Borders: Plant Foraging by Hawraman and Mukriyan Kurds in Western Iran

**DOI:** 10.3390/plants13071048

**Published:** 2024-04-08

**Authors:** Naji Sulaiman, Farzad Salehi, Julia Prakofjewa, Sofia Anna Enrica Cavalleri, Hiwa M. Ahmed, Giulia Mattalia, Azad Rastegar, Manijeh Maghsudi, Hawraz M. Amin, Ahmad Rasti, Seyed Hamzeh Hosseini, Abdolbaset Ghorbani, Andrea Pieroni, Renata Sõukand

**Affiliations:** 1 University of Gastronomic Sciences, Piazza Vittorio Emanuele II 9, 12042 Pollenzo, Italy; 2 Department of Environmental Sciences, Informatics and Statistics, Ca’ Foscari University of Venice, Via Torino 155, Mestre, 30170 Venezia, Italy; 3 RISTOLAB s.r.l., Via Caracciolo 88, 84068 Pollica, Italy; 4 World Food Forum Young Scientists Group (WFF YSG), 00153 Rome, Italy; 5 Bakrajo Technical Institute, Sulaimani Polytechnic University, Slemani 46001, Kurdistan Region, Iraq; 6 Institut de Ciència i Tecnología Ambientals (ICTA-UAB), Universitat Autònoma de Barcelona, 08193 Barcelona, Spain; 7 HKS Herbarium, Kurdistan Agricultural and Natural Resources Research and Education Center, AREEO, Sanandaj 6616936311, Iran; 8 Department of Anthropology, Tehran University, Tehran 1411713118, Iran; 9 Department of Chemistry, University of Pavia, Via Taramelli 12, 27100 Pavia, Italy; 10 Department of Chemistry, College of Science, Salahaddin University-Erbil, Erbil 44001, Iraq; 11 Department of Environmental Sciences and Policies, University of Milan, Via Celoria 2, 20133 Milan, Italy; 12 Department of Biology, Faculty of Science, University of Jiroft, Jiroft 7867155311, Iran; 13 Lärarhögskolan, University of Umea, 901 87 Umeå, Sweden; 14 Department of Medical Analysis, Tishk International University, Erbil 44001, Iraq

**Keywords:** ethnobotany, Fertile Crescent, Iran, Iraq, Middle East, Persian, wild food plants

## Abstract

Plant foraging is a millennia-old activity still practiced by many people in the Middle East, particularly in the Fertile Crescent region, where several socioeconomic, ecological, and cultural factors shape this practice. This study seeks to understand the drivers of plant foraging in this complex region characterized by highly diverse linguistic, religious, and cultural groups. Our study aims to document the wild plants used by Kurds in Western Iran, identify similarities and differences among Hawraman and Mukriyan Kurdish groups in Iran, and compare our findings with a previous study on the Hawramani in Iraq. Forty-three semi-structured in-depth interviews were conducted in Kurdish villages of Western Iran. The results revealed the use of 44 wild food plant taxa, their preparation, and culinary uses. Among the reported taxa, 28 plant taxa were used by Mukriyani, and 33 by Hawramani. The study revealed a significant difference between the Hawraman and Mukriyan regions in Iran, whereas there is a high similarity between Hawramani Kurds in Iran and Iraq. We found that the invisible cultural border carries more weight than political divisions, and this calls for a paradigm shift in how we perceive and map the distribution of ethnobotanical knowledge.

## 1. Introduction

Traditional ecological knowledge (TEK) is vital for local communities’ well-being and is an element of their cultural identity. TEK has been a focal point of research within the ethnobiological field, and considerable attention has recently been given to understanding the evolution of such knowledge over time and place [1]. Studies on cross-cultural comparison of ethnobotany among diverse ethnic or religious groups have a relatively robust history in the ethnobiology of the past four decades [2,3,4,5,6]. Ethnobotany along political borders has developed more recently [3,7,8,9] and was the main subject of the European Research Council-funded DiGe project [10]. The dynamics underpinning the evolution of food plant foraging and its embedded knowledge have been recently investigated in many studies. These studies underlined cross-cultural and cross-geographical comparative perspectives, paying particular attention to their evolution and the main drivers behind any observed changes within or between selected communities and areas [6,11,12,13,14,15,16,17]. Plant foraging and embedded gastronomic knowledge are essential expressions of local culture, shaped by the surrounding environment and historical context. Different cultures often develop specific recipes using distinct ingredients [18]. The underlying ecological knowledge is shaped by many factors, including sociocultural aspects such as religion, language, politics, governmental systems, and economic features [15,19,20]. In the comparisons conducted by the DiGe project [10], the cross-border differences in the wild food plant uses are often more pronounced than in the comparison between the cultural groups, differing in several aspects (ethnic, linguistic, and religious) but residing in the same country [12,18].

The social fabric of the Middle East, especially in the Fertile Crescent region, is highly diverse, with many linguistic, religious, and cultural groups, where Kurds represent an essential part of this mosaic [21]. Mesopotamia, and the regions around it, is a unique hotspot for biocultural diversity and for investigating patterns of traditional wild food plant foraging, considering that this area was home to the first Neolithic communities and has been, over millennia, a crossroad of different civilizations and cultures [16]. In the Middle East, Kurdish communities are separated by state borders, and one sub-group of these communities is represented by the Hawramani, which resides alongside the borders of Iran and the north-eastern part of the Kurdistan Region in Iraq. The region is unique in its flora and fauna and rich in cultural traditions, with numerous rituals such as the annual “PirShaliar” and “Komsai” festivals. The Cultural Landscape of the Hawraman district of the Kurdistan Province was recently added to the UNESCO World Heritage list because it exemplifies the millennia-long co-evolution of the semi-nomadic agropastoral Hawramani people [22]. Mukriyan is another Kurdish group that has its own cultural characteristics and is mainly present in the south of the Kurdistan province of Iran. However, despite this biocultural richness, studies on Kurdish ethnobotany published in international journals are sporadic [23]. Therefore, this study aims to celebrate such biocultural diversity and deepen the understanding of the factors influencing the evolution and circulation of local knowledge of wild plant use among the Hawramani and Mukriyani people of Iran. We mainly aim to:

1. Document the TEK related to using wild food plants by Mukriyan and Hawraman Kurds in western Iran.
2. Compare the wild plants mentioned by the Iranian Hawraman with a previously published study on Iraqi Hawraman.
3. Position our results within other studies conducted on cross-cultural ethnobiology and reflect on the differences and commonalities arising from cross-border territories to advance the hypothesis on how the circulation of local plant knowledge happens.

Based on the literature review and previous studies in the DiGe project [10] that aims to understand the mechanisms of changes in ethnobotanical knowledge, we hypothesize a homogeneous use of wild plants between Kurdish groups in Iran and a divergence with those living in Iraq.

## 2. Materials and Methods

### 2.1. Study Area

**Figure 1:**
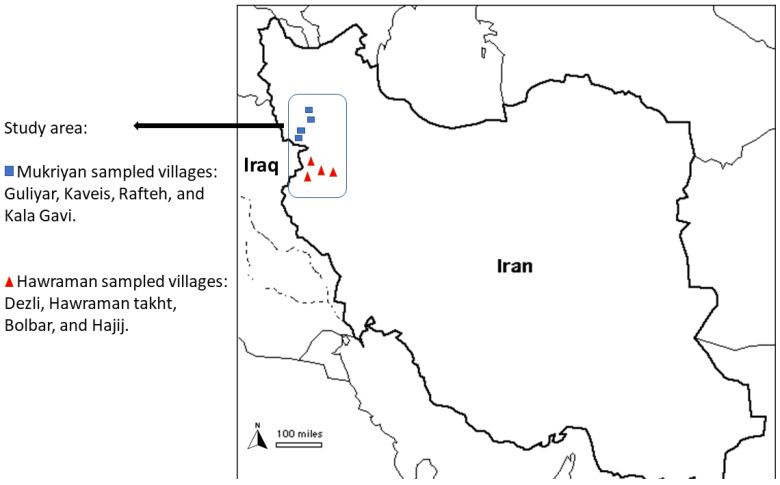
Study area map within the map of Iran.

The Mukriyan region (35°54′–36°52′ N and 44°45′–46°33′ E) is bounded to the north by Oshnavieh, Lake Urmia, and Maragheh, and to the south by the Kurdistan province (Figure 1) [24]. This territory shares natural borders with Iraqi-Kurdistan and Takab in the East Azerbaijan Province. The Mukriyan region lies at an altitude of 1400 to 1800 m.a.s.l. Mukriyan’s topography varies from hills to high-elevation mountains. The total population of the region is around 1,400,000. Mountain villages are typically inhabited by 50–200 people. Local people speak Kurdish Sorani, and most local people are bilingual in Persian, the country’s only official language. The climate in both Mukriyan and Hawraman regions is similar and classified as a humid continental climate with hot summers and cold winters, based of the Köppen Climate Classification.

**Figure 2:**
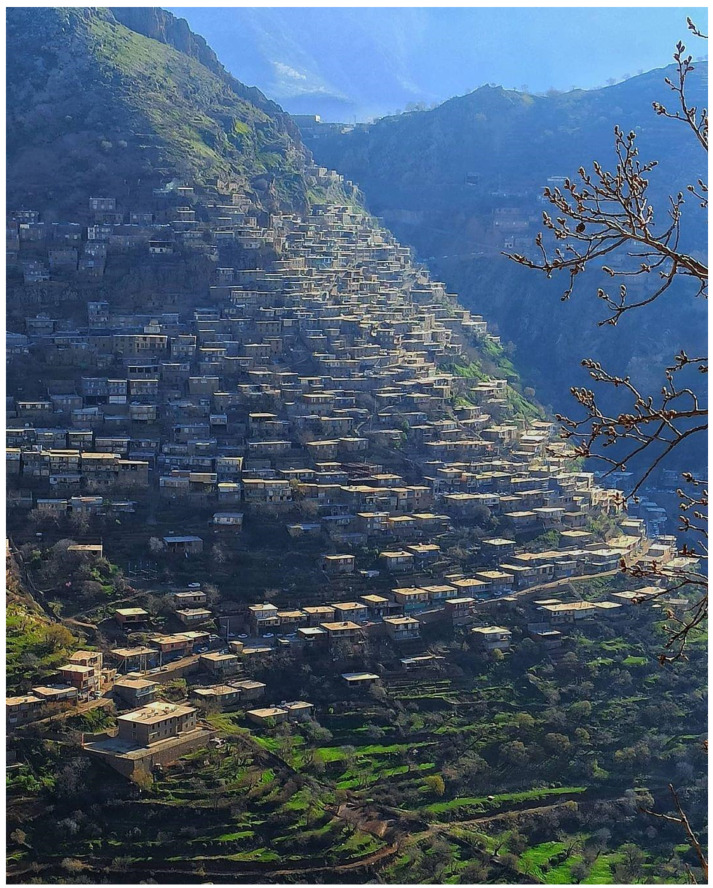
Uraman Takht, Kurdistan Province, in the Hawraman area, Iran (Photo credit: H.M. Ahmed).

Hawraman is a mountainous touristic region of Kurdistan, corresponding to the Kermanshah and Kurdistan provinces (Iran) and Halabja (Iraq). The mountains are at an altitude of 1270 to 3390 m.a.s.l. Due to the steep terrain, the cultivated area is not very large and quite steep, so, walnuts and other fruit trees form the main crop, alongside densely forested areas. Some houses in the area are built on the mountains slopes due to the lack of flat and stable land to build houses (Figure 2). Although there is no accurate census, the population of Hawraman is estimated to be around 750,000 persons.

Both regions share similarities in education, with schools operating to the national standard of Iran, with teaching conducted in Persian and Kurdish. The literacy level among those above 40 years of age reflects a period where education was not heavily emphasized, but it is now more common to find individuals who can read and write, thanks to educational policies in recent years. Regarding language, people in the Hawraman region speak Hawramani, which is a dialect of Goran that varies from place to place due to the geographical location; while Mukriyani, a dialect of Sorani, is used in Mukriyan in addition to Persian, which is the country’s only official language. Both populations predominantly practice Sunni Islam, with many in Hawraman adhering to the Naqshbandi order.

### 2.2. Data Collection and Data Analysis

We conducted the fieldwork in the spring of 2021 and 2022. Forty-three semi-structured in-depth interviews were performed in two regions of Western Iran. In our study, we covered a substantial distance, notably a 300-km journey from the Mukriyan region, starting at Mahabad, to Hawraman Takht, which required approximately six hours of driving time. In the mountainous region of Mukriyan, we conducted 22 interviews (13 women and 9 men) in the following mountainous villages of Mukriyan: Guliyar (1565 m.a.s.l), Kaveis (1415 m.a.s.l), Rafteh (1645 m.a.s.l), and Kala Gavi (1650 m.a.s.l) (Figure 1). In Hawraman, we engaged with 21 individuals (13 men and 8 women) across eight villages situated at altitudes ranging from 800 to 1650 m above sea level.

We used snowball methodology to identify the majority of middle-aged and elderly residents living in rural areas who may be traditional ecological knowledge holders [25]. After finishing each interview, participant was asked to recommend one or more of the local elderly people who are knowledgeable about wild food plants. The majority of study participants were elderly local people who spent most of their lives in the same village. The participants ranged between 40 and 80 years old, except four with an age range of 25 to 28. We interviewed in the Kurdish language Sorani dialect in Mukriyan, and Hawramani dialect among Hawramani people. Voucher specimens were collected, taxonomically identified, and deposited at the Herbarium, Forests and Rangelands Research Department, Kurdistan Agriculture and Natural Resources Research and Education Center, Sanandaj, Iran. We followed World Flora Online for the nomenclature of the reported species. We obtained informed verbal consent from each participant before conducting each interview and followed the Code of Ethics of the International Society of Ethnobiology [26]. Interviewees were first asked to provide general socioeconomic information such as gender, age, education, and occupation, residency location and origin (village, district, region), languages spoken (mother and father tongue). Afterwards, participants were asked about wild food plants they use now and in the past; we recorded local names and a description of traditional culinary preparations for each listed plant.

Data were organized and condensed in an Excel file, where the data were qualitatively and quantitatively analyzed. We calculated the frequency of citation for each species; in addition, we reported the frequency of citation of each species in each group of Hawramani and Mukriyani. To compare different ethnic groups and countries, the Jaccard similarity indices were determined using the method outlined by Cabrera-Meléndez et al. [27]. The formula used is the following: JI = (C/(A + B − C)) × 100. In this equation, “A” represents the total number of species or genera found in sample A, “B” indicates the total number in sample B, and “C” is the count of species or genera that are present in both sample A and B. We compared both studied communities and our results from Hawramani Iran with a previously published study on Hawramani Iraq [23], to obtain a broader understanding of the similarities and differences between these groups over cultural and long-standing state borders. The border between Iran and Iraq in Hawraman corresponds to the former border of the Ottoman Empire, which emerged from the Treaty of Zuhab (1639), in which the Ottoman–Persian frontier was formalized, with Iraq permanently ceded to the Ottomans. In addition, we compared previously published studies from the Middle East (e.g., [28,29,30,31,32,33]). The comparison findings among the considered groups were graphically represented using proportional Venn diagrams.

## 3. Results and Discussion

### 3.1. Ethnobotanical heritage of Hawramani and Mukriyani Kurds in Iran

Our study documented the use of 44 wild food plant taxa by Hawramani and Mukriyani Kurds in Western Iran (Table 1). The reported species belong to 40 genera and 20 botanical families. The most represented families were Apiaceae (eight species), Asteraceae and Lamiaceae (five species each), and Brassicaceae (four species). Among the reported taxa, 28 plant taxa were used by Mukriyani, and Hawramani used 33 taxa.

The results demonstrate a slight difference between the studied groups in terms of the number of used species as the Hawramani showed a higher diversity in wild food plants (33 plant species were used by the Hawramani, and 28 species were used by the Mukriyani). This could be due to the ecological context, as the Hawraman territory is characterized by a diversity of mountains, valleys and slopes, ports, belts, and rivers where many people collect wild food plants, while there are more plains dedicated to agricultural activity in the Mukriyan area. *Rheum ribes* was reported by 77% of our respondents while *Allium paradoxum*, *Mentha longifolia*, and *Tragopogon collinus* were mentioned by over 50% of the total interviewees. However, these species were not reported with the same saliency by both the Mukriyani and Hawramani groups (Table 2). For instance, none of the top-used plants in each sociocultural group are equally important to the other one. *A. paradoxum* was shown to be a critical species of the Hawramani culture (21 reports), while it was reported only three times among the Mukriyani. In contrast, *M. longifolia* was highly reported by the Mukriyani (23 reports) with six reports by the Hawramani. Similarly, *T. collinus* was also an essential species for the Mukriyani with 16 reports compared to five reports by the Hawramani. On the other hand, *R. ribes* was shown to be a significant species for both groups, mainly for the Hawramani (24 citations) and to a lesser degree for the Mukriyani with nine reports.

The results showed that Kurds in Iran have a slightly higher diversity in ethnobotanical knowledge compared to the Hawramani Kurds in Iraq when considering the results of both groups together [23]. However, by comparing each group separately, we find a roughly similar ethnobotanical diversity. On the other hand, we found that our reported diversity of used food plants is noticeably lower than that documented among Yazidis, Assyrians, and Muslim Kurds in Northern Iraq [32]. In addition, this diversity is significantly lower than the diversity recorded in the Mediterranean region of neighboring Syria [28], Turkey [31], and Armenia [33]. This could be attributed to some differences in the ecosystems as well as the slowly fading food and foraging heritage.

### 3.2. Transitional Knowledge over Cultural Borders: Differences and Similarities between Hawramani and Mukriyani Kurds in Iran

**Figure 3:**
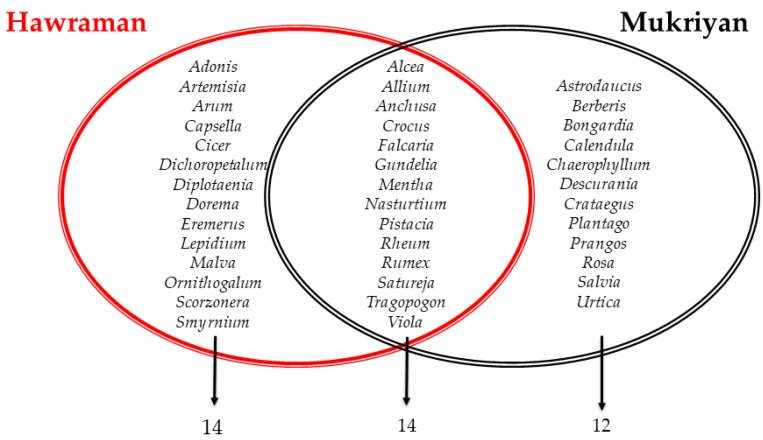
Reported wild plant genera by Hawraman and Mukriyan groups in Iran.

The comparison in the reported plant genera between Hawramani and Mukriyani groups in Iran showed an overlap in only 35% (14 genera) of the total number of genera (Figure 3). This can be considered a relatively low similarity as both groups belong to the same ethnic group (Kurd) within the same state.

**Figure 4:**
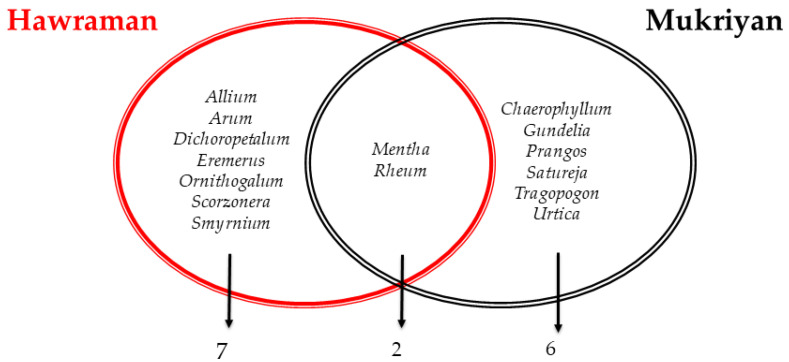
Top-quoted wild plant genera by Hawraman and Mukriyan groups in Iran.

This result was strengthened by comparing the top-quoted genera. Figure 4 shows only two top-quoted plant genera overlapping between the two groups. These overlapped genera (*Rheum* and *Mentha*) seem to be key species in Kurdish food culture, as they are also reported in other Kurdish regions in neighboring countries [16,23]. A recent study showed *Rheum* and *Mentha* are rich in active compounds that lead to curing health conditions. They are consumed as a snack food, especially *Rheum*, collected by local people during spring to make money for families in the high mountains and hills.

The genera reported solely by the Hawramani are mainly weeds and leafy vegetables such as *Arum*, *Lepidium*, and *Malva*. We observed the presence of some woody plants (shrubs and trees) among the plants reported solely by the Mukriyan such as *Berberis*, *Crataegus*, and *Rosa*. This can be interpreted by the differences in the origin of both groups and the dominant ecosystem in the land of origin (horticulturalism and pastoralism), as the Hawramani are considered to be descended from the people of Gilan near the Caspian Sea, who themselves are a mix of South Caucasians (Georgians, Armenians, etc.) and Persians which may potentially explain the Hawramani differences [34]. Moreover, despite the similarity in climate conditions between the two regions, elevation may have a slight impact on the availability of specific plant species, which may have played a minor role in the documented difference.

Results showed an almost identical similarity in plant vernacular names between the Hawramani in Iraq and Iran and to a lesser degree of similarity between the Hawramani and Mukriyani. We found slight differences in the mode of preparation and consumption of some species between the Hawramani in Iraq and Iran (e.g., *Allium* spp., *Anchusa* spp.). The Hawramani in Iraq showed a high interest in frying preparation methods compared to the preparation of soups in Iran; however, both showed consumption of wild plants as raw.

### 3.3. Transitional knowledge over State Borders: Differences and Similarities between Hawramani in Iran and Hawramani in Iraq

**Figure 5:**
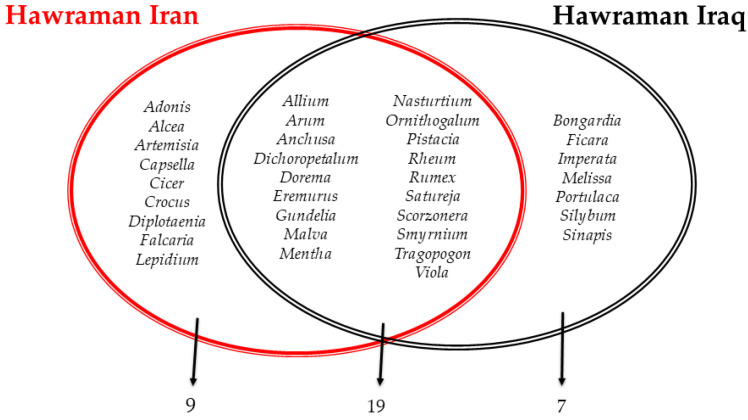
Reported wild plant genera by the Hawraman groups in Iran and Iraq.

The comparison between our results on Hawraman in Iran and the results of Pieroni et al. [23] on the Hawramani of Iraq, provided a broader understanding of the similarities and differences among the Kurdish communities in the Middle East (Figure 5). Both groups show significant overlap in the shared taxa (19 genera; JI = 54). This can be interpreted as some homogeneity within the same Hawramani groups despite the border division. The current border between Iraq and Iran reflects that between Persia and the Ottoman Empire and has thus been in place for four centuries. Furthermore, human movement over this border became very difficult during Saddam Hussain’s regime in Iraq and especially during the Iraq–Iran War in the second half of the 20th Century. However, our observations and the respondents’ reports confirmed that movements along this border still exist nowadays.

This unique similarity across state borders stands in contrast with the findings of Stryamets et al. [15], who highlighted the similarity between two ethnic groups (the case of the Hutsuls and Romanians) within the same country (Romania or Ukraine), while they found higher differences within groups across the border (Romanians living in Romania and Ukraine) and Belichenko et al. [35] for Seto people residing on the border between Estonia and Russia.

Kurds in Iran and Iraq show a considerable similarity in foraged plant genera with other parts of the Middle East. Most of the overlapped genera (e.g., *Allium*, *Anchusa*, *Malva*, and *Rumex*) are highly reported by other studies in the region such as Iraq [32], Syria [28], Armenia [33], Georgia [36,37], Pakistan and Afghanistan [30], Lebanon [38], Cyprus [39], and Turkey [31]. Moreover, some species seem culturally unique in the whole region of the Fertile Crescent and surrounding areas, such as *Gundelia* sp. Some other species are unique to specific areas and human groups in the region, such as *Arum* spp., which is widely used by Alawites in coastal Syria, Palestinians, Yazidis and Kurds in Iraq and Iran [23,28,29].

The currently obtained cross-border difference (JI = 54) is comparable with the high similarity (JI = 53) between Muslim and Kakai Kurds in Iraq [16]; while the dissimilarity between Hawraman and Mukriyan Kurds in Western Iran (JI = 35) resembles the difference between Muslim Kurds and Assyrians (J = 32) and Assyrians and Yazidis (JI = 34) in Iraq [32]. The cross-border JIs in DiGe studies [10] varied from 65 among Setos in Estonia and Russia, where the border was merely administrative until 30 years ago [35], to 55 among Hutsulsin Romania and Ukraine, where the solid border was established over 75 years before the study [8]. At the same time, the Polish–Belarus–Lithuanian borderlands ranged from 48 to 62, as in that once homogenous region, the borders shifted several times in the last 75 years [20]. Therefore, the cross-border JI between Hawraman Iran and Iraq stays within the results obtained in DiGe studies [10]. What differs is the proportional difference within the country, which contradicts all results of DiGe and our previous research on Ukraine [40].

The data presented in this comparative ethnobotanical study show that the differences in the use of wild food plants between the two Kurdish areas of Iran (Hawraman and Mukriyan) are greater than those between the Iraqi and Iranian Kurdish Hawraman. However, this border existed for four centuries, as the former long-standing border between the Ottoman Empire and Persia. This border was most possibly very porous until the 1980s of the 20th century, with the arising war between Iraq and Iran. The critical differences between the two Kurdish groups in Iran could be explained considering the tribal nature of the Kurdish umbrella societies, which have shaped kinship relations over centuries and are also reinforced in this case by different languages (Hawramani is not even considered a Kurdish language by most linguistic taxonomies) [41,42]. The more homogenous results between the two sides of the borders instead account for the robust social exchanges and circulation of knowledge between the borders. In 2016, this article’s corresponding author could still observe very regular contact between these two communities, with daily visits of Iranian Hawramani male smugglers to Hawramani Iraqi villages and their relatives after sunset. This, in turn, can be explained by the very distinctive Hawramani culture. In earlier work, we cited historical sources, which agree on a horticulturalists-driven origin of Hawramanis, who migrated centuries ago from NW Iran to their nowadays territory (contrary to a presumed pastoralist origin of all other Kurds from the central Iranian Plateau) [23]. Therefore, our current results suggest that the “invisible” cultural border between Hawraman and Mukriyan Kurds appears more robust than that linked to the effects of the Iraqi/Iranian political borders. A similar picture was observed among Friulians and Slovenes of NE Italy [43], while opposite findings emerged in diverse previous studies; one was conducted among Hutsulst and Boykos in SW Ukraine [40]; one explained the overall outcomes of the food ethnobotanical dataset of the DiGe project conducted at the Eastern fringe of the former Soviet Union borders (where the robust effect of Soviet centralizing policies made plant knowledge on the non-Soviet side of the former border very remarkable) [11]. These divergent results can be explained by looking at the very different degrees of centralization operated by the respective nation-states in their peripheral areas during the past century.

## 4. Conclusions

Ethnobotanical data from our study show that cultural borders in the study area are relevant to articulating more or less dense exchanges of folk food plant knowledge. The juxtaposition of ethnobotanical studies with cross-cultural and cross-border analysis adds a layer of complexity to our understanding of traditional ecological knowledge (TEK) circulation. The traditional practice of plant foraging, deeply rooted in local cultural identities in the study area, often transcends normative state borders, challenging conventional notions of territorial boundaries. While geopolitical borders may stand as physical boundaries on maps, the circulation of TEK demonstrates that knowledge flows with a fluidity that defies man-made constraints.

On the other hand, the current study shows the cultural differences among the Kurdish communities even within the same state. These “invisible” cultural borders between the Hawramani and other Kurds appear to exert a stronger influence than the effects of political borders. This challenges prevailing assumptions and prompts a reevaluation of how we conceptualize and map ethnobotanical knowledge evolution and distribution.

Integrating cross-cultural and geopolitics into the ethnobotanical discourse requires a nuanced understanding of the factors influencing knowledge transfer. Despite the geopolitical separation, the regular contacts observed between Iranian Hawramani communities and their Iraqi counterparts highlight the resilience of cultural ties that persist over centuries. These interactions, often facilitated by local dynamics and cultural commonalities, emphasize the need to generate “ethnobotanical maps” to better reflect the actual pathways of knowledge flow.

In light of these considerations, this study contributes to a growing body of literature challenging the rigidity of normative borders in ethnobotanical research. The conclusion that the “invisible” cultural border carries more weight than political divisions calls for a paradigm shift in how we perceive and map the distribution of traditional ecological knowledge. By acknowledging the fluidity and adaptability of TEK across geopolitical landscapes, ethnobotanical studies can better capture the rich tapestry of cultural practices and plant-foraging heritage that transcend long-standing nation-state borders.

Moreover, the presented results could significantly impact fostering a revitalization of foraging as an essential strategy for local food security and sovereignty. This particular trajectory of valorizing wild food plants in local gastronomy and food specialty products could be extremely promising for further promoting sustainable biocultural heritage-centered rural tourism. Foraging could also play a significant role in rural sustainable development, since, as a practice deeply rooted in traditional knowledge, it has been a critical survival strategy for peripheral communities for centuries. Incorporating foraged foods into local diets can enhance nutritional diversity and reduce dependence on external food sources. Additionally, the recorded foraged plant ingredients could offer some economic opportunities, i.e., wild-harvested products could be sold locally or in regional city markets. Eventually, foraging practices, since often deeply intertwined with biodiversity and cultural heritage, could help local communities to preserve wild habitats and cultural identities by strengthening the intergenerational transfer of nature knowledge. Future studies are needed to investigate the nutritional value of the reported species, as this may be vital for the food security of the local communities.

## Figures and Tables

**Table 1:** Wild food plants used by our Hawramani and Mukriyani participants in Western Iran, in addition to a comparison with Hawramani in Iraq.

Scientific Name; Family; Voucher Code	Local Name(s)	Part Used	Local Food Use	Frequency of Citation (FoC) by both Mukriyan and Hawraman in Iran	Reported by Mukriyani Kurds in Iran (FoC)	Reported by Hawramani Kurds in Iran (FoC)	Reported by Hawramani Kurds in Iraq [23]
*Adonis aestivalis* L.; Ranuncolaceae HSK52	Seiraian	Young aerial parts	Boiled, fried	2	No	Yes (2)	No
*Alcea kurdica* (Schltdl.) Alef.; Malvaceae; HSK44	Harmalei	Leaves and flowers	Soups, tea	5	Yes (3)	Yes (2)	No
*Allium iranicum* (Wendelbo) Wendelbo; Amaryllidaceae; HSK48	Kanival, Knivali	Aerial parts	Seasoning bread (mixed with the dough before baking it)	5	No	Yes (5)	No
*Allium jesdianum* Boiss. and Buhse; Amaryllidaceae; HSK09	Surkabna	Whole plant	Soup	15	Yes (4)	Yes (11)	Yes
*Allium paradoxum* (M. Bieb.) G. Don; Amaryllidaceae; HSK40	Pichek	Leaves	Raw, soups, seasoning (esp. bread)	24	Yes (3)	Yes (21)	Yes
*Anchusa italica* Retz.; Boraginaceae; HSK19	Gozirvan, Gozorvaneh	Flowers	Seasoning, fried, tea	9	Yes (3)	Yes (6)	Yes
*Artemisia annua* L. Asteraceae; HSK57	Barzalang	Leaves	Tea, seasoning yoghurt	1	No	Yes (1)	No
*Arum maculatum* L. and possibly other *Arum* spp. Araceae; HSK50	Kardu, Khaz	Aerial parts	Soups, stewed	7	No	Yes (7)	Yes
*Astrodaucus orientalis* (L.) Drude; Apiaceae; HSK13	Zizra manda	Stems	Raw	5	Yes (5)	No	No
*Berberis vulgaris* L.; Berberidaceae; HSK33	Zereshk	Fruits	Soups	1	Yes (1)	No	No
*Bongardia chrysogonum* (L.) Spach; Berberidaceae; HSK54	Gable	Young inflorescences	Stewed	3	Yes (3)	No	Yes
*Calendula officinalis* L.; Asteraceae; HSK22	Hamisha bahara	Flowers	Raw, fried, jam	3	Yes (3)	No	No
*Capsella bursa-pastoris* (L.) Medik.; Brassicaceae; HSK53	Paklachaka	Leaves	Soups, fried	2	No	Yes (2)	No
*Chaerophyllum macrospermum* (Willd. ex Schult.) Fisch. and C.A.Mey. ex Hohen.; Apiaceae; HSK01	Mandok	Aerial parts	Soups, pickled	13	Yes (13)	No	No
*Cicer kermanense* Bornm.; Fabaceae; HSK20	Nokashoana	Flowers	Soups	1	No	Yes (1)	No
*Crataegus azarolus* L.; Rosaceae; HSK15	Jevzh	Fruits	Snack	1	Yes (1)	No	No
*Crocus haussknechtii* (Boiss. and Reut. ex Maw) Boiss. and possibly other *Crocus* spp.; Iridaceae; HSK23	Kifok, Pishok	Corms	Snack, roasted, added to milk	7	Yes (3)	Yes (4)	No
*Descurainia sophia* (L.) Webb ex Prantl; Brassicaceae; HSK29	Shivaran	Leaves	Tea, drunk cold	1	Yes (1)	No	No
*Dichoropetalum aromaticum* (Rech.f.) Pimenov and Kljuykov; Apiaceae; HSK37	Baraza	Aerial parts	Raw, rarely seasoning	7	No	Yes (7)	No
*Diplotaenia damavandica* Mozaff., Hedge and Lamond; Apiaceae; HSK55	Gzlki	Roots	Seasoning	1	No	Yes (1)	No
*Dorema aucheri* Boiss.; Apiaceae	Bana	Young aerial parts	Raw, fried	4	No	Yes (4)	Yes
*Eremurus spectabilis* M.Bieb.; Asphodelaceae; HSK39	Khuzha, Khuzhe	Leaves	Raw, soups, seasoning	17	No	Yes (17)	Yes
*Falcaria vulgaris* Bernh.; Apiaceae; HSK07	Kaziakha	Leaves	Raw, soups, stewed	8	Yes (4)	Yes (4)	No
*Gundelia tournefortii* L. Asteraceae; HSK02	Kinger	Stems	Soups, fried	13	Yes (9)	Yes (4)	Yes
*Lepidium persicum* Boiss.; Brassicaceae; HSK28	Hazba koilai, hazba kevialla	Aerial parts	Consumed fresh, boiled, seasoning, tea	6	No	Yes (6)	No
*Malva* spp.; Malvaceae	Tollaka	Leaves	Cooked	4	No	Yes (4)	Yes
*Mentha arvensis* L.; Lamiaceae; HSK17	Nana	Leaves	Seasoning	2	Yes (2)	No	No
*Mentha longifolia* (L.) L.; Lamiaceae; HSK04	Pinga	Aerial parts	Raw, seasoning	29	Yes (23)	Yes (6)	Yes
*Mentha requienii* Benth.; Lamiaceae; HSK36	Karas	Aerial parts	Raw, tea	8	No	Yes (8)	No
*Nasturtium officinale* W.T.Aiton; Brassicaceae; HSK27	Kuzala, Kashala	Aerial parts	Raw, soups	11	Yes (7)	Yes (4)	Yes
*Ornithogalum caudatum* Aiton and possibly other *Ornithogalum* spp.; Asparagaceae; HSK41	Ruzka, Gelakh	Bulbs	Stewed	6	No	Yes (6)	Yes
*Pistacia atlantica* Desf.; Anacardiaceae; HSK49	Kaswan	Unripe fruits and soft stems	Seasoning, pickled	6	Yes (3)	Yes (3)	No
*Plantago major* L.; Plantaginaceae; HSK25	Jia barkholah	Leaves	Snack	1	Yes (1)	No	No
*Prangos aricakensis* Behçet and Yapar; Apiaceae; HSK14	Biza	Leaves	Snack, seasoning cheese, soups	14	Yes (14)	No	No
*Rheum ribes* L.; Polygonaceae; HSK03, HSK42	Revas	Stems	Snack, jams, soups, fried, tea	33	Yes (9)	Yes (24)	Yes
*Rosa canina* L.; Rosaceae; HSK34	Bagh	Flowers	Jam	1	Yes (1)	No	No
*Rumex* spp.; Polygonaceae; HSK35	Tirshoka, Tisho	Leaves and stems	Raw, soups	11	Yes (5)	Yes (6)	Yes
*Salvia bracteata* Banks and Sol.; Lamiaceae; HSK24	Jia chai	Aerial parts	Tea	2	Yes (2)	No	No
*Satureja* spp.; Lamiaceae	Jatrah, Kashmah	Leaves	Seasoning	16	Yes (10)	Yes (6)	Yes
*Scorzonera* sp.; Asteraceae; HSK21	Shing	Aerial parts	Raw, fried	10	No	Yes (10)	Yes
*Smyrnium cordifolium* Boiss.; Apiaceae; HSK46	Gnur, Ninur	Stems, leaves	Raw, seasoning (also black tea)	10	No	Yes (10)	Yes
*Tragopogon collinus* DC. and possibly other *Tragopogon* spp.; Asteraceae; HSK21	Halakok, Asping	Leaves	Raw, fried	21	Yes (16)	Yes (5)	Yes
*Urtica* spp.; Urticaceae; HSK05	Gazgask	Young aerial parts	Soups, stewed, tea	9	Yes (9)	No	No
*Viola cornuta* L.; Violaceae; HSK43	Vanavshah	Flowers	Tea, jams	8	Yes (2)	Yes (6)	No

**Table 2:** Most reported plants by each sociocultural group (number of reports are placed between brackets).

Hawramani	Mukriyani
*Rheum ribes* (24)	*Mentha longifolia* (23)
*Allium paradoxum* (21)	*Tragopogon collinus* (16)
*Eremurus spectabilis* (17)	*Prangos aricakensis* (14)

## Data Availability

The data that support the findings of this study are presented in the article.
